# A dataset of orthogonal polygon-derived maze environments for path planning benchmarking

**DOI:** 10.1016/j.dib.2026.112841

**Published:** 2026-05-17

**Authors:** Nguyen Kieu Linh

**Affiliations:** Posts and Telecommunications Institute of Technology, Hanoi, Vietnam

**Keywords:** Orthogonal polygons, Maze generation, Path planning, Benchmark dataset, Computational geometry, Motion planning

## Abstract

This paper presents a dataset of grid-based maze environments constructed from orthogonal polygonal structures. The orthogonal polygons are generated using a constructive generation framework. Building upon these polygonal structures, the present work further constructs maze environments by transforming polygons into grid-based representations, automatically generating start–goal configurations, and producing associated benchmarking data. The dataset covers a wide range of geometric complexities by varying the number of polygon vertices. Each maze instance is represented on a discrete grid, where polygon boundaries are treated as obstacles and interior regions as navigable space. The data are provided in a structured format, including orthogonal polygon representations, grid-based mazes, visualization images, and metadata, allowing direct use in computational experiments without additional preprocessing. Benchmark results are included for several classical path planning algorithms, including Breadth-First Search (BFS), Dijkstra’s algorithm, A*, Probabilistic Roadmaps (PRM), Rapidly-exploring Random Trees (RRT-Connect), and RRT*. For each instance, performance indicators such as success rate, path length, and execution time are reported to illustrate basic usage of the dataset. In addition, an extended stochastic evaluation is provided, where sampling-based methods are executed over five independent runs and RRT is further analyzed under a convergence-based stopping criterion, offering a more comprehensive view of variability and solution refinement. The dataset can support benchmarking and comparative evaluation of path planning algorithms, as well as studies on the influence of geometric structure on navigation tasks. In addition, it may be used in related application contexts such as procedural maze generation in games, simulation environments with controlled spatial complexity, and as a source of data for learning-based approaches to navigation, where both maze structures and algorithm-generated trajectories are available. The data generation pipeline and associated resources are provided to facilitate reproducibility and further reuse.

Specifications Table

**Table:** 

Subject	Computer Sciences
Specific subject area	Computational geometry and path planning benchmark datasets
Type of data	Grid-based maze data; polygon vertex data; JSON files; images (visualizations) Raw and processed data
Data collection	The data were generated using a constructive algorithm for orthogonal polygon generation implemented in Python. The polygon generation procedure is based on a constructive orthogonal polygon generation framework described in [1]. Polygon instances with a prescribed number of vertices were produced and subsequently transformed into grid-based maze representations. The transformation process maps polygon boundaries and exterior regions to obstacles, while the interior of each polygon is treated as navigable space. Each maze is represented on a discrete grid with automatically determined resolution based on the polygon complexity. Start and goal points were assigned algorithmically for each instance using a two-sweep Breadth-First Search (BFS) procedure to approximate distant reachable pairs within the maze. All data were generated programmatically without the use of external measurement instruments or experimental devices.
Data source location	Institution: Posts and Telecommunications Institute of Technology (PTIT) City: Hanoi Country: Vietnam Latitude and longitude: Not applicable (synthetic dataset)
Data accessibility	Repository name: Zenodo Data identification number: https://doi.org/10.5281/zenodo.19990818 Direct URL to data: https://zenodo.org/records/19990818 [2] Code repository: https://github.com/linhnk2109/orthogonal-maze-benchmark Instructions for accessing: Publicly accessible without restriction.
Related research article	A companion research article describing the polygon generation methodology is provided in [1].

## 1 Value of the Data

• The dataset provides a collection of maze environments derived from orthogonal polygonal structures with varying numbers of vertices, enabling controlled variation in geometric complexity.
• The data can be directly reused as benchmark instances for evaluating path planning algorithms, including both graph-based and sampling-based methods in grid-based environments.
• The standardized data format, together with automatically generated start–goal configurations, supports reproducible experiments and facilitates integration into existing simulation frameworks.
• The dataset allows systematic evaluation of algorithm performance under structured conditions, including environments with varying spatial characteristics such as narrow passages and complex layouts.
• In addition, the underlying polygonal data may be used for benchmarking geometric algorithms, such as orthogonal hull computation and minimum-area rectilinear convex hull computation.
• The data may be of interest to researchers in computational geometry, robotics, artificial intelligence, and related fields, and may also be applied in procedural environment generation and algorithm testing.
• Unlike many existing path planning datasets that emphasize uniformly distributed obstacles or game-like terrains, this dataset focuses on orthogonal polygon-derived mazes with narrow passages. This structural property creates challenging environments for path planning algorithms, particularly in cases involving constrained navigation.
• To improve the reliability of performance evaluation, the dataset includes multi-trial benchmarking results for stochastic algorithms such as PRM, RRT-Connect, and RRT*. Reported metrics are averaged over multiple independent runs, allowing for more stable and reproducible comparisons.
• In addition to standard evaluations, the dataset supports the analysis of asymptotic behavior in sampling-based methods. Specifically, benchmark results and trajectory visualizations are provided for an optimized RRT* variant using a convergence-based termination criterion, enabling the study of solution refinement beyond the first feasible path.

## 2 Background

**Fig. 1:**
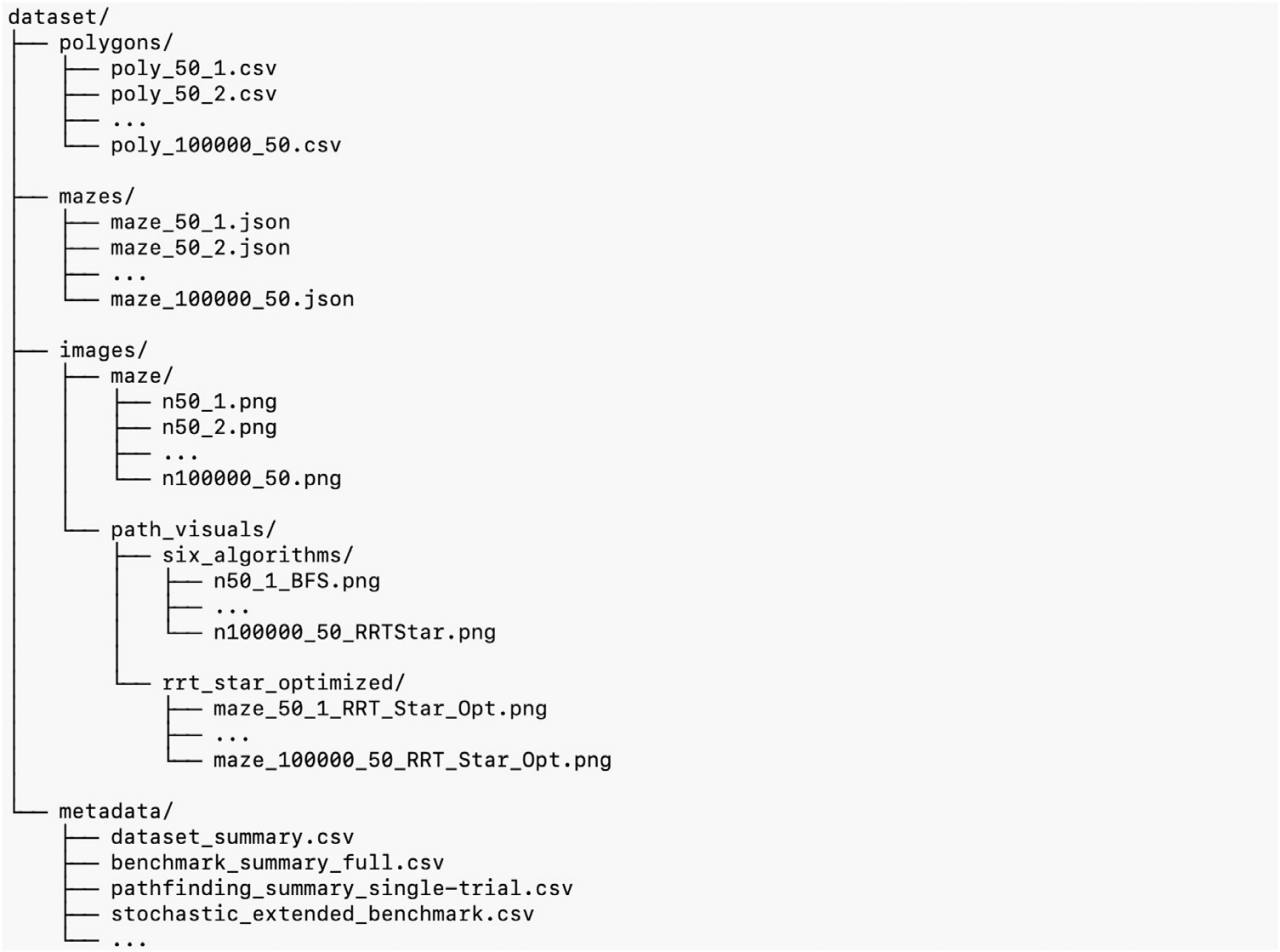
Directory structure of the dataset, showing polygon data, corresponding maze instances, visualization outputs, and metadata files.

**Fig. 2:**
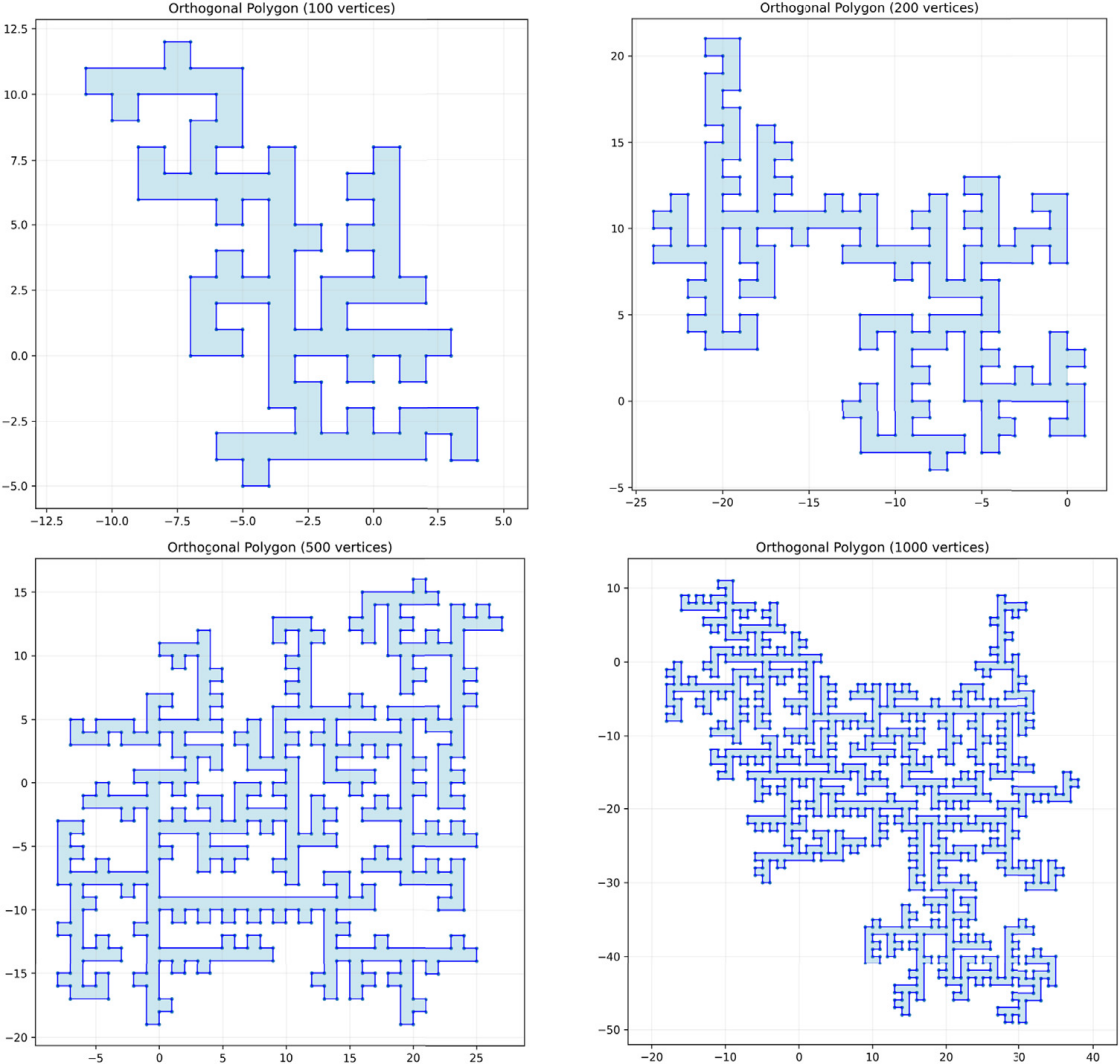
Representative orthogonal polygon instances generated with varying numbers of vertices (100, 200, 500, and 1000), demonstrating the scalability of the constructive generation process and the resulting structural complexity.

**Fig. 3:**
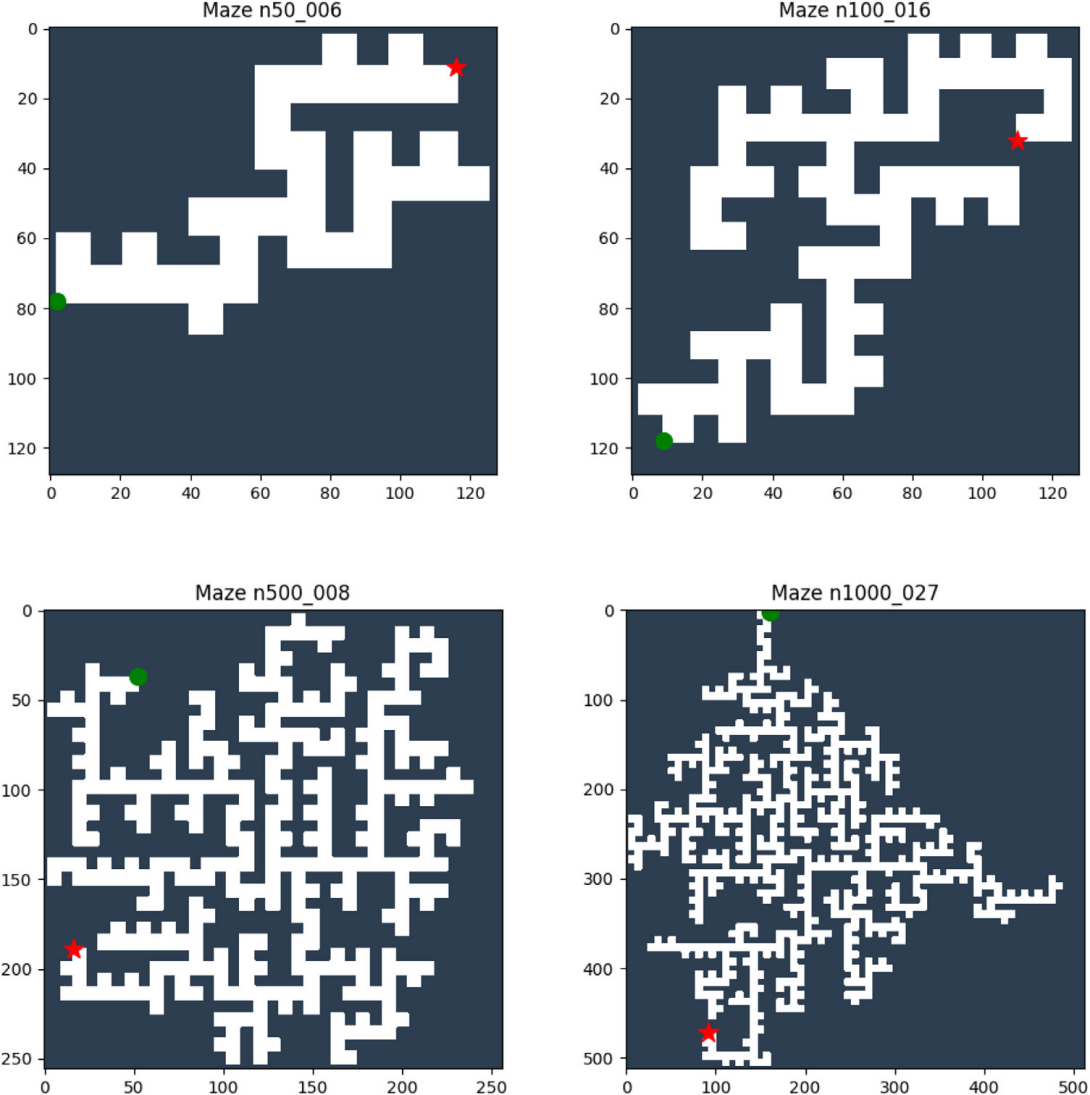
Representative grid-based maze instances generated from orthogonal polygons with varying numbers of vertices (50, 100, 500, and 1000). Free space and obstacles are shown in white and dark regions, respectively, with start (green) and goal (red) positions indicated.

The generation of structured geometric data plays an important role in the evaluation of computational algorithms. Orthogonal polygonal structures are commonly used in computational geometry as test instances due to their well-defined properties and suitability for a range of geometric problems constructive algorithm for generating such polygons with a prescribed number of vertices has been developed in previous work by the authors [1]. That work focuses on the design, correctness, and efficiency of the algorithm, and includes benchmarking on representative geometric algorithms Fig. 1, Fig. 2, Fig. 3.

The dataset presented in this article is derived from the polygonal instances produced by that algorithm. The polygons are further transformed into grid-based maze environments by interpreting their boundaries as obstacles and their interiors as navigable regions. This transformation allows the reuse of geometric structures in a different experimental setting.

The purpose of compiling this dataset is to provide a collection of structured instances that can be used for evaluating path planning algorithms under controlled geometric conditions. By varying the number of vertices in the underlying polygons, the dataset includes instances with different levels of structural complexity.

## 3 Data Description

The dataset is organized into a structured directory format to facilitate access and reuse (Fig. 1). It consists of orthogonal polygon data, corresponding maze representations, and metadata files [2]. The main directory is organized as follows.

### 3.1 Polygon data (datasets/polygons/)

This section describes the organization and structure of the polygon data stored in the dataset/polygons/. The dataset covers a wide range of geometric complexities by considering 20 different vertex counts, ranging from 50 to 100,000 vertices. For each vertex count, 50 polygon instances are generated, resulting in a total of 1000 orthogonal polygons and 1000 corresponding maze instances.

Each CSV file stores a single orthogonal polygon and follows a consistent naming scheme: poly_{*n*}_{*i*}.csv, where:

• *n* denotes the number of vertices of the polygon, which reflects its geometric complexity (e.g., 50, 100, 500, 1000),
• *i* denotes the index of the instance within the group of polygons with *n* vertices, formatted using zero-padding (e.g., 1, 2, 3,…).
• For example, the file poly_100_3.csv contains the vertex coordinates of the third polygon in the group with 100 vertices.

The polygon data are generated using a constructive algorithm for orthogonal polygon generation. The process begins from a simple initial configuration and incrementally modifies the structure through local geometric transformations.

During generation, the following properties are maintained:

• Orthogonality: all edges are parallel to either the horizontal or vertical axis,
• Simplicity: the polygon does not contain self-intersections (Fig. 2).

Once the desired number of vertices *n* is reached, the resulting polygon is exported directly to a CSV file.

Each CSV file follows a simple and consistent format. An example is shown below:

**Table:** 

The structure is interpreted as follows:

• The first row is a header indicating the two-dimensional Cartesian coordinates (x, y),
• Each subsequent row corresponds to a vertex of the polygon, represented as a pair of floating-point values,
• The vertices are listed in sequential order along the polygon boundary (typically in counter-clockwise order).

This ordering defines the edges implicitly: each pair of consecutive vertices forms an edge, and the last vertex is connected back to the first to form a closed polygon.

### 3.2 Maze data (datasets/mazes/)

The dataset/mazes/ directory contains grid-based maze environments derived from orthogonal polygonal structures. Each file is stored in JSON format and follows a consistent naming convention of the form maze_{*n*}_{*i*}.json, where *n* denotes the number of vertices of the original polygon and *i* denotes the index of the instance within the corresponding group.

For example, the file maze_100_3.json represents the third maze instance generated from polygons with 100 vertices. Each JSON file stores a dictionary containing four main fields.

An example structure is shown below:

**Table:** 

The maze data are obtained through a geometric transformation process. Each polygon is first scaled and embedded into a square grid of appropriate size to preserve its structural properties. The grid is then constructed by classifying each cell according to its spatial relation with the polygon: cells inside the polygon are labeled as free space, while cells on the boundary or outside the polygon are treated as obstacles. Start and goal configurations are selected from the free space of the grid, with a preference for positions that are relatively far apart to provide meaningful navigation tasks (Fig. 3).

Each JSON file contains four main fields: grid_size, start, goal, and grid. The grid_size specifies the dimension of the square grid, the start and goal fields define the initial and target positions in grid coordinates, and the grid field represents a binary occupancy matrix. In this representation, cells are encoded as 0 for free space and 1 for obstacles. The data format is designed to allow direct use in computational experiments without additional preprocessing.

### 3.3 Visualization data (datasets/images/)

The dataset/images/ directory provides visual representations of the generated maze environments and the corresponding path planning results. It consists of two subdirectories: maze/ and path_visuals/.

The dataset/images/maze/ subdirectory contains visualizations of the maze environments before applying any path planning algorithms. The files are named using the format n{*n*}_{*i*}.png, which is consistent with the identifiers of the corresponding polygon and maze data files. Each image is generated by rendering the binary grid representation using a predefined colormap, where free space is shown in white and obstacles are shown in dark gray. The start and goal positions are overlaid on the image as markers, with the start point indicated by a green circle and the goal point indicated by a red star. These visualizations provide an intuitive understanding of the geometric structure of the maze. This directory contains 1000 images, each corresponding to a maze environment with annotated start and goal positions.

The dataset/images/path_visuals/six_algorithms subdirectory contains visualizations of path planning results obtained from various algorithms, including BFS, Dijkstra, A*, PRM, RRT, and RRT*. The file names follow the format n{*n*}_{*i*}_{Algorithm}.png, where the suffix indicates the algorithm used. Each image is generated by overlaying the computed trajectory on the corresponding maze map. The final path connecting the start and goal positions is displayed as a prominent red curve, while, for sampling-based methods, the exploration graph or tree is shown using faint blue lines. In addition, each image includes a title summarizing key information such as the algorithm name, path length, and execution time. These visualizations illustrate the behavior of different algorithms and support qualitative comparison across methods. Under the dataset and experimental setup described in this paper, this directory contains 6000 images illustrating the computed paths and, where applicable, intermediate exploration structures.

The directory dataset/images/path_visuals/rrt_star_optimized contains supplementary visualizations of the optimized RRT* algorithm, comprising a total of 502 PNG files generated under the dataset and experimental setup described in this paper. These images are included only for instances where a valid path is successfully found during the extended multi-trial evaluation. Each file, named in the format maze_{n}_{i}_RRT_Star_Opt.png, overlays the optimized trajectory on the corresponding grid map, with the final path highlighted in red. Intermediate exploration trees are omitted to emphasize the converged solution. The image title summarizes the averaged results over five trials, including the maze identifier, average path length (Avg Len), and average execution time (Avg Time). These visualizations provide qualitative insight into the convergence behavior of RRT* under extended optimization.

### 3.4 Metadata (datasets/metadata/)

The dataset/metadata/ directory provides tabular summaries of the dataset and supports efficient access, querying, and large-scale analysis without the need to process individual data files. It contains some main CSV files.

• The file dataset_summary.csv provides an overview of all instances in the dataset and establishes links between polygon data, maze representations, and initialization parameters. Each row corresponds to a single instance and includes the following information: an instance identifier (instance_id), the number of vertices (num_vertices), references to the corresponding polygon and maze files (poly_file, maze_file), and the start and goal coordinates. The instance identifier serves as a unique key that enables consistent referencing across different components of the dataset, while the inclusion of start and goal positions allows direct use in path planning experiments without additional parsing.
• The file benchmark_summary_full.csv contains benchmark results obtained by applying several classical path planning algorithms, including BFS, Dijkstra, A*, PRM, RRT, and RRT*, to the maze instances. Each row records the performance of a specific algorithm on a given instance, identified by a reference to the corresponding maze. The recorded metrics include a success indicator (success), the path length (path_length), and the execution time in seconds (time_sec). These measurements provide a basis for comparing algorithm performance in terms of feasibility, solution quality, and computational cost.
• The file pathfinding_summary_single-trial.csv summarizes the results from benchmark_summary_full.csv, where each reported metric is averaged over 50 instances for each value of *n*.
• The file stochastic_extended_benchmark.csv presents extended benchmark results for stochastic algorithms (PRM, RRT-Connect, and RRT*), where each metric is averaged over 5 independent runs. It also includes results from an optimized RRT* variant to support analysis of asymptotic convergence behavior.

## 4 Experimental Design, Materials and Methods

### 4.1 Orthogonal polygon generation

Orthogonal polygons in the dataset are generated using a constructive algorithm defined on the integer lattice $Z^{2}$ [1]. The procedure takes as input a target number of vertices *n* (even, *n* ≥4) and incrementally constructs a simple orthogonal polygon *P* with exactly *n* vertices.

The generation process begins with an initial configuration that depends on the value of *n*. If n ≡0 (mod 4), the algorithm initializes *P* as a unit square with four lattice vertices. Otherwise, an *L*-shaped orthogonal polygon with six lattice vertices is used as the starting configuration. In both cases, the polygon is embedded in the integer grid, and a set U is maintained to store all lattice points that lie in the interior or on the boundary of the current polygon.

The polygon is then expanded iteratively until the required number of vertices is reached. At each iteration, a convex vertex *a* = (*x, y*) of the current polygon *P* is selected uniformly at random. A direction l∈{east,west,north,south}is then chosen, and the neighboring lattice point *a*' in that direction is considered. If the edge [*a, a*'] belongs to the boundary of *P*, a local expansion operation is performed.

The expansion is realized through an *L*-shaped construction that replaces the selected vertex with a sequence of new vertices. Depending on whether *a*' is itself a vertex or lies on an edge of *P*, two slightly different configurations of the *L*-shape are used. In both cases, a fixed pattern of lattice points is generated, and a set of new vertices {$b_{1}$, $b_{2}$, $b_{3}$, $b_{4}$, $b_{5}$} is inserted in place of the original vertex a, increasing the total number of vertices by four. The polygon boundary is updated while preserving the counter-clockwise ordering of vertices.

To ensure validity, the algorithm checks that the newly introduced lattice points do not intersect with existing points in *U*, except for those being replaced. If the expansion would introduce overlaps or violate simplicity, the operation is discarded and the previous configuration is retained. Otherwise, the polygon *P* and the lattice set *U* are updated accordingly.

This iterative process continues until the number of vertices satisfies |*V*(*P*)| = *n*. The resulting polygon is guaranteed to be simple (non-self-intersecting) and orthogonal, with all edges aligned to the coordinate axes.

In addition to the incremental construction, an alternative generation strategy based on orthogonal convex hull construction is also implemented for large-scale instances. In this approach, a set of points is sampled on a circle and partitioned into arcs, and the orthogonal convex hull of the resulting point set is computed to produce a simple orthogonal polygon with a prescribed number of vertices. This provides an additional mechanism for generating structurally diverse instances.

The final polygon is represented as an ordered sequence of vertices along its boundary and is exported to a CSV file for subsequent processing.

### 4.2 Grid construction and maze representation

Each generated orthogonal polygon is transformed into a grid-based maze representation through a rasterization process. Given a polygon defined in continuous coordinates, the polygon is first scaled and embedded into a square grid. The grid resolution is determined adaptively based on the number of vertices (*n*) of the polygon to preserve structural details, ensure dataset reproducibility, and avoid rasterization-induced topological collapse (i.e., the unintended fusion of adjacent narrow corridors). Specifically, the grid resolution is determined using a step-wise allocation strategy based on the polygon vertex count n. The grid size is set to 128×128 for polygons with n ≤ 100, and increased to 256×256 for 100 < n ≤500. For higher geometric complexities (n > 500), the grid resolution is strictly capped at 512×512 to maintain computational tractability and avoid excessive memory consumption. This fixed upper bound is a key factor underlying the topological degradation phenomena observed in the benchmark results. In particular, when highly complex polygons (e.g., n = 100,000) are rasterized onto the constrained 512×512 grid, narrow structural components and sub-pixel corridors may disappear during discretization, thereby altering the navigable topology of the resulting maze environment.

The scaling process maps the polygon from its original bounding box to the grid domain while preserving its geometric proportions. A padding margin is introduced to ensure that the polygon is fully contained within the grid. The rasterization is performed using a polygon filling procedure, where all grid cells located inside the polygon are assigned the value 0 (free space), and all remaining cells, including the boundary and exterior, are assigned the value 1 (obstacles). This approach ensures a consistent and connectivity-preserving conversion from continuous geometry to a discrete grid representation.

### 4.3 Start-goal configuration

For each maze instance, start and goal positions are optionally generated to support path planning tasks. These positions are selected from the set of free cells in the grid. A two-phase breadth-first search procedure is used to identify pairs of points that are relatively far apart in terms of path distance. Specifically, a random free cell is first selected, and a breadth-first search is performed to find a distant reachable cell. A second search is then performed from this point to identify another cell that is far in terms of shortest-path distance. The resulting pair of points is used as the start and goal configuration.

The start and goal coordinates are stored in grid coordinates and are provided as part of the maze data. These configurations enable direct use of the dataset in path planning experiments. In applications that do not require navigation tasks, this information can be omitted, and the grid representation alone can be used.

### 4.4 Path planning algorithms used for benchmarking

To illustrate the usage of the dataset, six pathfinding algorithms were implemented and evaluated. The algorithms are grouped into two categories: discrete grid-based methods and sampling-based methods. All distance computations and collision checks were implemented consistently to ensure comparable evaluation conditions.

### 4.5 Discrete grid-based algorithms

These algorithms operate directly on the binary grid representation of the maze.

Breadth-First Search (BFS) [3]: BFS was implemented using a 4-connected grid (up, down, left, right). It computes shortest paths in terms of the number of grid steps and serves as a baseline method without heuristic guidance.

Dijkstra’s algorithm [4]: Dijkstra’s algorithm was implemented on an 8-connected grid, allowing both orthogonal and diagonal movements. Orthogonal moves have unit cost, while diagonal moves have cost $\sqrt{2}$. A corner-cutting constraint was applied to prevent invalid diagonal transitions through obstacle boundaries.

A* algorithm [5]: A* uses the same movement model and constraints as Dijkstra’s algorithm, with the addition of a Euclidean distance heuristic. It provides a reference for shortest-path computation with heuristic guidance on grid-based environments.

### 4.6 Sampling-based algorithms

To evaluate sampling-based methods, the grid environment was treated as a continuous two-dimensional space. Sampling was restricted to navigable regions to ensure that generated configurations lie within free space.

Probabilistic Roadmap (PRM) [6]: The number of samples was scaled proportionally to the number of free cells in each instance, with predefined upper and lower bounds. Each sample was connected to a fixed number of nearest neighbors to form a roadmap graph. Paths were computed using Dijkstra’s algorithm on the constructed graph.

RRT-Connect [7]: A bidirectional tree-based method was used, growing two trees from the start and goal configurations. The maximum number of iterations was scaled with the size of the environment. A fixed step size was used to control the expansion of the trees.

RRT* [8]: An optimized variant of RRT [9] was implemented with reduced step size and neighborhood radius to adapt to narrow grid structures. A small goal bias was applied during sampling. To limit computation time, the algorithm was terminated shortly after the first feasible path was found.

**Fig. 4:**
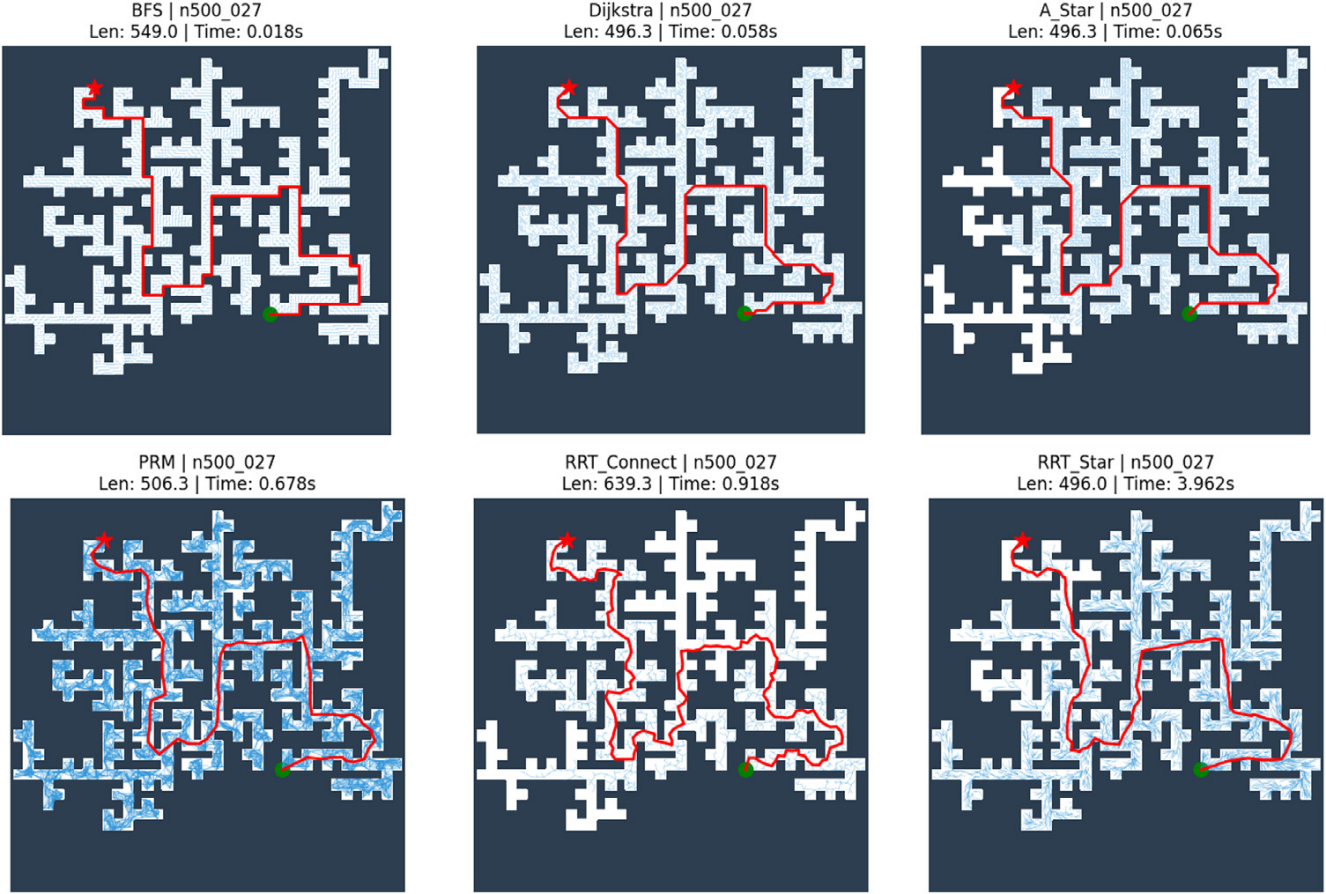
Visualization of path planning results on a representative maze instance (n = 500) using BFS, Dijkstra, A*, PRM, RRT-Connect, and RRT*. The computed paths are shown in red, with exploration structures, path lengths, and execution times indicated.

All algorithms are implemented under consistent conditions using the same grid representation, collision checking procedure, and start-goal configurations to ensure comparability across different planning approaches. The results are provided in a structured format and include path length, execution time, and success indicators for each instance. Fig. 4 presents representative path planning outcomes on a maze instance with *n* = 500, illustrating differences in exploration behavior and resulting trajectories across grid-based and sampling-based algorithms.

### 4.7 Benchmark results

All experiments were conducted on a MacBook Pro (14-inch, Nov 2023) with an Apple M3 Pro chip, 18 GB memory, running macOS Sonoma 14.3. All algorithms were implemented in Python and executed under the same software environment to ensure fair and consistent benchmarking conditions.

**Fig. 5:**
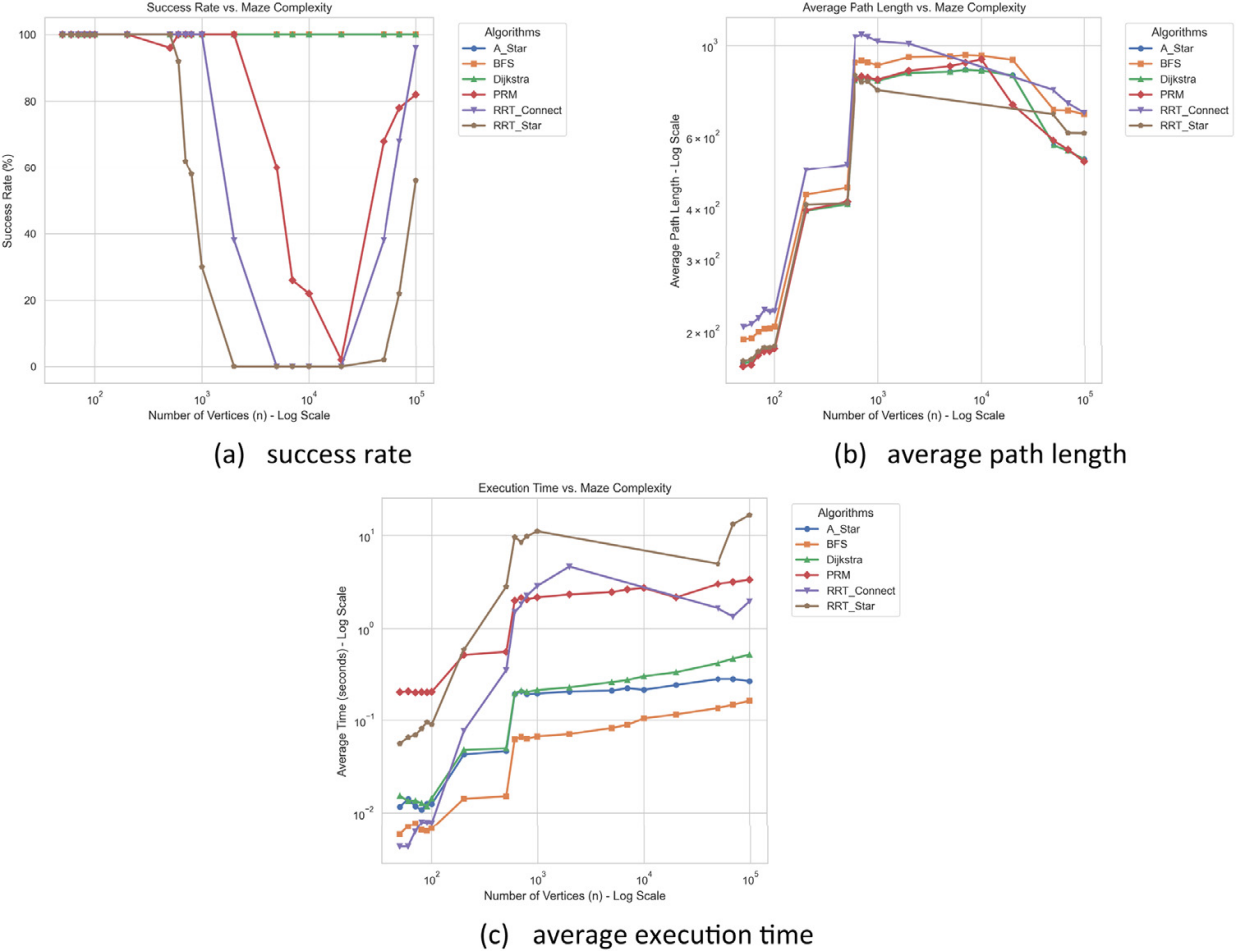
Benchmark results of six path planning algorithms across maze instances of increasing geometric complexity, showing (a) success rate, (b) average path length, and (c) average execution time as functions of the number of vertices.

The benchmark results illustrate the performance of six path planning algorithms across maze instances of increasing geometric complexity. Summary statistics are reported in Table 1, Table 2, Table 3, while the corresponding trends are visualized in Figs. 5. In this initial baseline evaluation, all algorithms were executed for a single trial per instance. Crucially, for the stochastic sampling-based group (PRM, RRT-Connect, and RRT*), this single-trial execution serves as a preliminary baseline to observe their raw, single-shot spatial exploration behaviors without statistical aggregation. Specifically, within this sampling-based group, an early-termination condition was applied to RRT* (halting immediately after finding the first feasible path) to establish a baseline for its initial spatial exploration speed prior to any asymptotic optimization.

**Table 1:** Summary of success rates (%) for path planning algorithms across maze instances of increasing geometric complexity.

n	A*	BFS	Dijkstra	PRM	RRT-Connect	RRT*
**50**	100.0	100.0	100.0	100.0	100.0	100.0
**60**	100.0	100.0	100.0	100.0	100.0	100.0
**70**	100.0	100.0	100.0	100.0	100.0	100.0
**80**	100.0	100.0	100.0	100.0	100.0	100.0
**90**	100.0	100.0	100.0	100.0	100.0	100.0
**100**	100.0	100.0	100.0	100.0	100.0	100.0
**200**	100.0	100.0	100.0	100.0	100.0	100.0
**500**	100.0	100.0	100.0	96.0	100.0	100.0
**600**	100.0	100.0	100.0	100.0	100.0	92.0
**700**	100.0	100.0	100.0	100.0	100.0	62.0
**800**	100.0	100.0	100.0	100.0	100.0	58.0
**1000**	100.0	100.0	100.0	100.0	100.0	30.0
**2000**	100.0	100.0	100.0	100.0	38.0	0.0
**5000**	100.0	100.0	100.0	60.0	0.0	0.0
**7000**	100.0	100.0	100.0	26.0	0.0	0.0
**10,000**	100.0	100.0	100.0	22.0	0.0	0.0
**20,000**	100.0	100.0	100.0	2.0	0.0	0.0
**50,000**	100.0	100.0	100.0	68.0	38.0	2.0
**70,000**	100.0	100.0	100.0	78.0	68.0	22.0
**100,000**	100.0	100.0	100.0	82.0	96.0	56.0

- RRT*: To evaluate its raw spatial exploration capability prior to any asymptotic optimization, an early-termination condition was applied (halting after finding the first feasible path). The step size was tightly restricted to 2.0 units with a 5.0-unit search radius for local rewiring, preventing invalid connections across thin obstacles. The goal-biasing probability was set at 5% to reduce collision-checking overhead in highly constrained corridors.

**Table 2:** Average path length of six path planning algorithms for maze instances with varying numbers of vertices.

n	A*	BFS	Dijkstra	PRM	RRT_Connect	RRT*
**50**	167.38	191.34	167.38	164.25	205.36	169.32
**60**	168.44	192.8	168.44	165.63	208.87	170.79
**70**	177.09	199.82	177.09	174.77	215.92	178.63
**80**	181.44	203.16	181.44	178.81	226.31	182.72
**90**	182.3	203.62	182.3	179.02	223.37	182.76
**100**	183.9	205.66	183.9	181.6	224.81	184.28
**200**	394.15	431.48	394.15	395.15	496.27	407.65
**500**	408.8	449.06	408.8	414.67	512.4	410.94
**600**	823.31	908.2	823.31	826.42	1046.13	847.19
**700**	838.8	918.34	838.8	841.29	1063.3	815.47
**800**	831.95	909.92	831.95	834.56	1049.69	817.9
**1000**	821.3	895.14	821.3	826.22	1023.23	779.26
**2000**	856.51	936.7	856.51	867.61	1010.21	N/A
**5000**	863.32	940.66	863.32	890.63	N/A	N/A
**7000**	872.57	947.82	872.57	907.23	N/A	N/A
**10,000**	868.41	944.2	868.41	924.84	N/A	N/A
**20,000**	845.81	922.16	845.81	718.36	N/A	N/A
**50,000**	572.74	696.94	572.74	588.24	779.74	680.73
**70,000**	555.39	695.6	555.39	559.09	724.31	613.48
**100,000**	528.98	681.0	528.98	522.51	686.92	612.54

Note: 'N/A' (Not Applicable) indicates that the algorithm failed to find a valid path for any instance at this complexity level.

**Table 3:** Average execution time (seconds) of six path planning algorithms for maze instances with varying numbers of vertices.

n	A*	BFS	Dijkstra	PRM	RRT_Connect	RRT*
**50**	0.01164	0.0058	0.01537	0.20173	0.00428	0.05599
**60**	0.01419	0.00706	0.01344	0.206	0.00429	0.06572
**70**	0.0118	0.00762	0.0136	0.19972	0.00618	0.06926
**80**	0.01081	0.00648	0.01281	0.20141	0.0079	0.08115
**90**	0.01261	0.00631	0.0118	0.20003	0.00784	0.09567
**100**	0.01253	0.00673	0.01443	0.20308	0.00773	0.09007
**200**	0.04281	0.01425	0.04796	0.5083	0.077	0.59079
**500**	0.04657	0.01517	0.04978	0.54945	0.34828	2.82594
**600**	0.19236	0.06233	0.19613	1.99461	1.50333	9.59172
**700**	0.20494	0.06658	0.21175	2.14829	1.80798	8.46585
**800**	0.19204	0.06316	0.20252	2.06288	2.25281	9.80053
**1000**	0.19428	0.06708	0.21203	2.16414	2.87078	11.12427
**2000**	0.20456	0.07113	0.22707	2.32548	4.64045	N/A
**5000**	0.20922	0.08262	0.25825	2.46506	N/A	N/A
**7000**	0.22232	0.08959	0.27205	2.6272	N/A	N/A
**10,000**	0.21363	0.10497	0.29886	2.72727	N/A	N/A
**20,000**	0.24053	0.11576	0.33	2.15806	N/A	N/A
**50,000**	0.27931	0.13569	0.41422	3.02302	1.65675	4.95935
**70,000**	0.27964	0.14722	0.46279	3.16422	1.34537	13.20905
**100,000**	0.26436	0.16263	0.51209	3.33871	1.96271	16.60882

Note: 'N/A' (Not Applicable) indicates that the algorithm failed to find a valid path for any instance at this complexity level.

To ensure the benchmark is highly reproducible and computationally tractable across varying scales, the default parameters for the sampling-based group were dynamically configured. For the initial baseline evaluation (single-trial):

- PRM (Probabilistic Roadmap): The number of sampled configurations was dynamically set to 15% of the available free space area (num_samples = len(free_cells) * 0.15), bounded between 1000 and 15,000 to prevent memory overflow in large instances. The connection radius (k-nearest neighbors) was set to 15 to ensure robust local connectivity through narrow passages.

- RRT-Connect: The step size was configured to 4.0 units, and the maximum iteration limit (max_iter) was dynamically bounded between 10,000 and 50,000, scaled as 50% of the grid area.- RRT*: To evaluate its raw spatial exploration capability prior to any asymptotic optimization, an early-termination condition was applied (halting after finding the first feasible path). The step size was tightly restricted to 2.0 units with a 5.0-unit search radius for local rewiring, preventing invalid connections across thin obstacles. The goal-biasing probability was set at 5% to reduce collision-checking overhead in highly constrained corridors.

Fig. 5(a) and Table 1 (success rate) highlight a clear distinction between algorithm classes. Grid-based methods maintain a 100% success rate across all tested instances, reflecting their completeness on discrete grids. In contrast, sampling-based algorithms experience a significant drop in success rate as the complexity increases, particularly in the presence of narrow passages. Nevertheless, for very large instances, a partial recovery in success rate can be observed for some methods.

Fig. 5(b) and Table 2 (path length) indicate that Dijkstra and A* consistently produce shorter paths compared to BFS, as expected from weighted shortest-path formulations. Sampling-based algorithms generally produce longer or more variable paths, especially at moderate complexity levels, due to their stochastic nature and reliance on sampled connections. However, for larger instances, the differences in path length across algorithms become less pronounced.

Fig. 5(c) and Table 3 (execution time) show that grid-based algorithms (BFS, Dijkstra, and A*) exhibit relatively stable and predictable growth in runtime as the number of vertices increases. Among them, BFS consistently achieves the lowest execution time, while Dijkstra and A* incur slightly higher computational costs due to weighted transitions and heuristic evaluations. In contrast, sampling-based methods (PRM, RRT-Connect, and RRT*) demonstrate higher variability in runtime. In particular, RRT* shows significantly larger execution times, reflecting the additional overhead associated with path optimization.

For instances with very large numbers of vertices (n > 20, 000), an observable change in the structure of the grid-based maze representations may occur. Due to the fixed grid resolution (up to 512×512), fine geometric details of highly complex polygons, such as narrow corridors or thin obstacle boundaries, may not be fully preserved during the rasterization process.

As a result, some maze instances may exhibit more connected free space regions compared to their original polygonal structures. This effect is a consequence of the discretization process and should be taken into account when interpreting benchmarking results, particularly for sampling-based path planning algorithms.

To better account for the stochastic nature of sampling-based methods and to provide a more complete evaluation of RRT*, we conducted an additional set of experiments. In this extended evaluation, PRM, RRT-Connect, and RRT* were each run for five independent trials per instance, and the reported metrics were averaged across runs (see the supplementary file *stochastic_extended_benchmark.csv*).

**Fig. 6:**
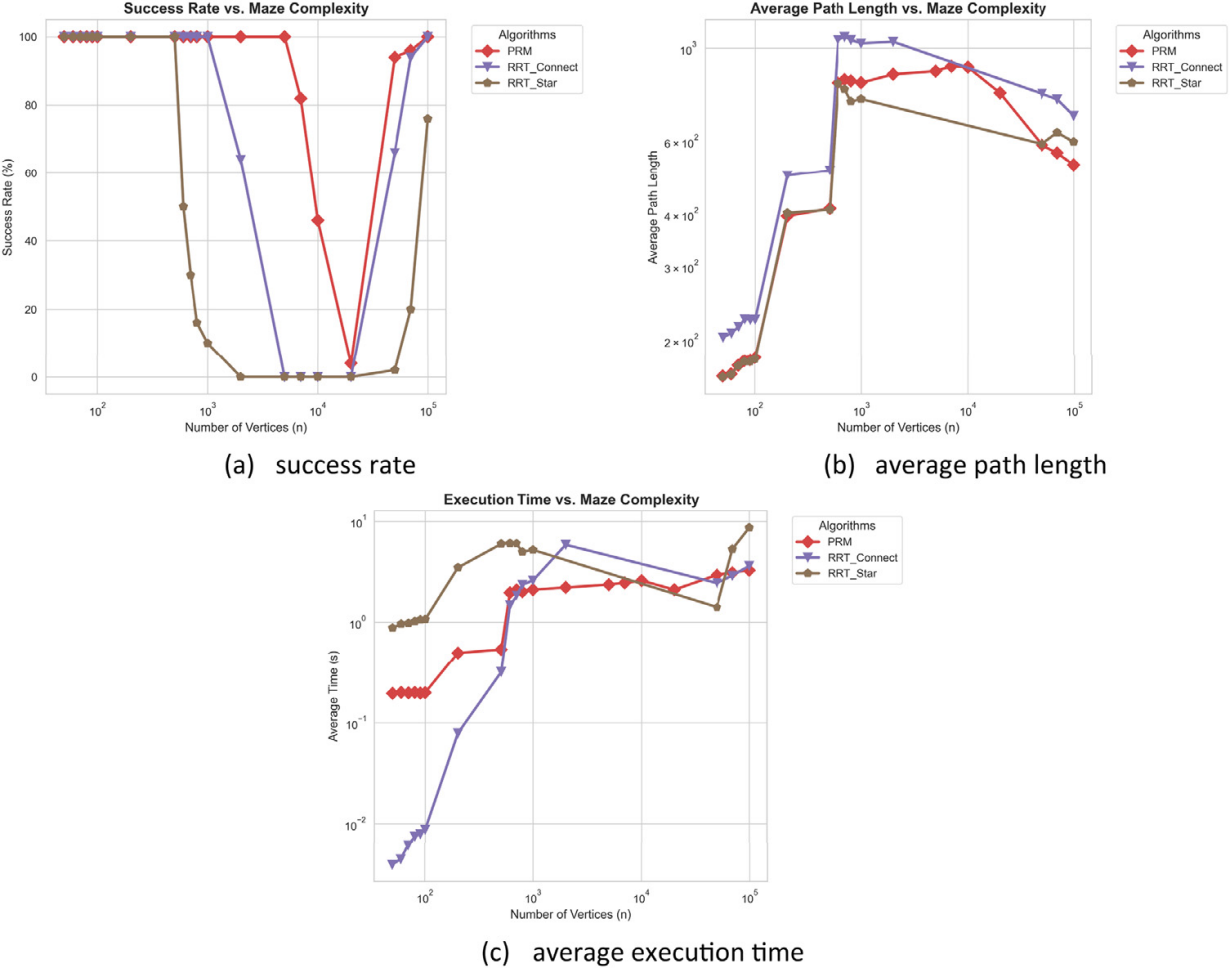
Statistical performance of PRM, RRT-Connect, and RRT* averaged over 5 independent trials per instance under extended stochastic evaluation.

The corresponding results are illustrated in Fig. 6(a–c) and summarized in Table 4, Table 5, Table 6, highlighting the averaged performance of all methods and the extended optimization behavior of RRT*.

**Table 4:** Summary of success rates (%) for PRM, RRT-Connect, and RRT* averaged over 5 independent trials per instance.

n	PRM	RRT_Connect	RRT_Star	n	PRM	RRT_Connect	RRT_Star
**50**	100.0	100.0	100.0	**800**	100.0	100.0	16.0
**60**	100.0	100.0	100.0	**1000**	100.0	100.0	10.0
**70**	100.0	100.0	100.0	**2000**	100.0	64.0	0.0
**80**	100.0	100.0	100.0	**5000**	100.0	0.0	0.0
**90**	100.0	100.0	100.0	**7000**	82.0	0.0	0.0
**100**	100.0	100.0	100.0	**10,000**	46.0	0.0	0.0
**200**	100.0	100.0	100.0	**20,000**	4.0	0.0	0.0
**500**	100.0	100.0	100.0	**50,000**	94.0	66.0	2.0
**600**	100.0	100.0	50.0	**70,000**	96.0	94.0	20.0
**700**	100.0	100.0	30.0	**100,000**	100.0	100.0	76.0

**Table 5:** Average path length for PRM, RRT-Connect, and RRT* averaged over 5 independent trials per instance.

n	PRM	RRT_Connect	RRT_Star	n	PRM	RRT_Connect	RRT_Star
**50**	164	203.5	163.21	**800**	836.25	1047.19	745.98
**60**	165.9	208.22	165.23	**1000**	827.16	1026.99	756.59
**70**	174.1	215.76	173.51	**2000**	866.52	1035.97	N/A
**80**	178.7	225.05	177.59	**5000**	882.01	N/A	N/A
**90**	179.4	224.52	177.72	**7000**	905.46	N/A	N/A
**100**	181.7	224.64	180.17	**10,000**	901.08	N/A	N/A
**200**	395.8	495.8	402.63	**20,000**	782.13	N/A	N/A
**500**	412.9	510.02	410.17	**50,000**	587.21	778.58	590.61
**600**	826.4	1045.57	824.05	**70,000**	562.3	755.89	629.54
**700**	841.6	1064.26	798.06	**100,000**	526.76	690.41	597.65

Note: 'N/A' (Not Applicable) indicates that the algorithm failed to find a valid path for any instance at this complexity level.

**Table 6:** Average execution time (seconds) for PRM, RRT-Connect, and RRT* averaged over 5 independent trials per instance.

n	PRM	RRT_Connect	RRT_Star	n	PRM	RRT_Connect	RRT_Star
**50**	0.1945	0.00394	0.87927	**800**	2.00901	2.3588	4.98244
**60**	0.1996	0.00446	0.96501	**1000**	2.10709	2.60058	5.20936
**70**	0.1969	0.00612	0.98039	**2000**	2.2115	5.86096	N/A
**80**	0.1994	0.00746	1.02023	**5000**	2.36676	N/A	N/A
**90**	0.196	0.00793	1.06026	**7000**	2.45573	N/A	N/A
**100**	0.1985	0.0088	1.06926	**10,000**	2.5939	N/A	N/A
**200**	0.497	0.07876	3.48578	**20,000**	2.10245	N/A	N/A
**500**	0.5374	0.31781	5.97038	**50,000**	2.9561	2.44266	1.4116
**600**	1.9678	1.48577	6.05194	**70,000**	3.09644	2.90519	5.30135
**700**	2.1167	1.83402	6.04039	**100,000**	3.28135	3.62124	8.62084

Note: 'N/A' (Not Applicable) indicates that the algorithm failed to find a valid path for any instance at this complexity level.

For RRT*, the early termination condition was removed. Instead, we used a convergence-based stopping criterion, where the algorithm terminates if no improvement in path cost is observed over 1500 consecutive iterations. This setup allows RRT* to further refine its solution through rewiring, providing a more representative view of its performance under extended optimization. Example trajectories generated under this setting are available in the directory *images/rrt_star_optimized/*.

Compared to the single-run setting (Table 1, Table 2, Table 3) and the multi-run evaluation averaged over five independent runs (Table 4, Table 5, Table 6), PRM and RRT-Connect show generally consistent results across both settings. In contrast, RRT* exhibits more noticeable differences due to the incorporation of a convergence-based stopping criterion. However, under this extended setup, RRT* does not improve in terms of success rate; instead, it tends to produce shorter paths, closer to the optimal solutions (e.g., A* and Dijkstra), at the cost of increased execution time.

## Limitations

The dataset is generated from synthetic orthogonal polygonal structures and therefore may not fully capture the diversity and irregularity of real-world environments. The maze representations are derived from discretized grid mappings, which may introduce resolution-related artifacts depending on the selected grid size. Although grid resolution is adjusted based on polygon complexity, very fine geometric details may still be approximated.

The dataset focuses exclusively on two-dimensional environments with axis-aligned (orthogonal) structures. As a result, it does not include non-orthogonal geometries or higher-dimensional configuration spaces commonly encountered in more complex motion planning problems.

Start and goal configurations are generated automatically using a distance-based procedure to ensure long navigation paths. While this provides consistent and challenging instances, it may not reflect all possible task scenarios.

In addition, the benchmark results included in the dataset are limited to a selected set of classical path planning algorithms under fixed parameter settings. Different parameter choices or algorithmic variants may lead to different outcomes.

## Ethics Statement

The authors confirm that they have read and comply with the ethical requirements for publication in *Data in Brief*. The present work does not involve human subjects, animal experiments, or data collected from social media platforms. The dataset is entirely generated through computational methods without the use of personal, sensitive, or identifiable data, and therefore does not require ethical approval.

## CRediT Author Statement

**Nguyen Kieu Linh:** Conceptualization, Methodology, Software, Data curation, Validation, Visualization, Writing - original draft, Writing - review & editing.

## Data Availability

ZenodoA Dataset of Orthogonal Polygon-Derived Maze Environments for Path Planning Benchmarking (Original data).
